# Effects of Resistance Training With Constant, Inertial, and Combined Loads on Muscle Power and Strength Output

**DOI:** 10.3389/fphys.2021.709263

**Published:** 2021-11-25

**Authors:** Saša Đurić, Olivera M. Knezevic, Vedrana Sember, Ivan Cuk, Aleksandar Nedeljkovic, Maja Pajek, Dragan M. Mirkov

**Affiliations:** ^1^Faculty of Sports, University of Ljubljana, Ljubljana, Slovenia; ^2^Institute for Medical Research, University of Belgrade, Belgrade, Serbia; ^3^The Research Center, Faculty of Sport and Physical Education, University of Belgrade, Belgrade, Serbia; ^4^Faculty of Physical Education and Sports Management, Singidunum University, Belgrade, Serbia

**Keywords:** exercise, resistance, performance, biomechanics, muscle strength

## Abstract

The aim of this study was to investigate the resistance-specific gains in muscle power and strength (1RM) following the training of maximum bench-press throws (BPT) against constant, inertial, and combined resistance. Forty-eight male participants (age 20.5 ± 2.0 years) were randomly assigned to the constant, inertial, combined resistance, or control group. Participants underwent 8 weeks of training of BPT against the loads that corresponded to the different effects of mass of 40 kg (∼50% of 1RM). The gains in average and maximum power, and 1RM were significant in all experimental groups (*P* < 0.01), but not in the control group (*P* > 0.1). Relative gains in the average (26.3 ± 9.8%) and maximum power (25.2 ± 9.8%) were larger than that in the 1RM (mean 7.2 ± 6.9%; both *P* < 0.001). The gains in the average (*F*_4, 66_ = 6.0; *P* < 0.01) and maximum power (*F*_4, 66_ = 4.7; *P* < 0.01) were higher when tested against the training-specific resistance than when tested against the remaining two resistance types. Differences in 1RM among experimental groups were not significant (*P* = 0.092). The most important and rather novel finding of the study is that the training against the weight and inertial resistance, and their combination results in resistance-specific gains in muscle power, although the overall gains muscle strength and power remain comparable across the training protocols.

## Introduction

Both the training and testing methods related to muscle force and power-producing capacities have been in the focus of research for decades (Kaneko, 1983; Vandewalle et al., 1987; Jaric, 2015). Regarding the magnitude of training load, it has been argued that the most effective one for maximizing mechanical power output ranged from 40 to 70% for bench press and bench press throw (BPT) depending on gender, level of training, and many other factors (Mayhew et al., 1997; Baker et al., 2001; Cronin et al., 2001; Izquierdo et al., 2001, 2002). Regarding the type of applied resistance, the most frequently applied external resistance originates from a lifted mass of either the moved body segments, or additional load, or both. In such exercises, the exerted muscle force needs to overcome the weight and inertia of the lifted mass that both act along the same line when lifting an object vertically. Note that the resistance originating from weight provides a constant force, while the inertial resistance changes over time proportional to the acceleration. The effect of only weight could be produced either by a slow lifting where the acceleration (and, therefore, inertia) is negligible or by adding long and extensively stretched rubber bands pulling downward mimicking a constant force (Markovic and Jaric, 2007; Leontijevic et al., 2013; Djuric et al., 2016). Conversely, a combination of added weights and stretched rubber bands pulling upward with a similar force (Leontijevic et al., 2013; Markovic et al., 2013; Djuric et al., 2016) or performing rapid swings and push-offs in a predominantly horizontal direction (Liu et al., 2011; Samozino et al., 2012) can result in a severely reduced effect of weight, while the inertial resistance remains unchanged. Interestingly, although the weight type and inertia type of external resistance result in a very different pattern and magnitude of the agonist and antagonist muscle activities (Gottlieb, 1989), the specific effects of training against those two types of resistances have been largely neglected in the literature. Specifically, most of the previous studies have been conducted with the addition of external weights that inevitably increase both the weight and inertia, thus not allowing for differentiating between their effects (Newton et al., 1997; McBride et al., 2002; Drinkwater et al., 2005). Furthermore, the effects of those types of resistances on the muscle power and force capacities have been mostly investigated in movements where gains in power and force were often accompanied by resistance-specific adaptations of movement pattern such as in vertical jumping (Lyttle et al., 1996; Markovic et al., 2011, 2013; Zivkovic et al., 2017a). Namely, the changes in movement pattern are known to alter the power and force outputs, thus confounding the effect of the applied training procedures (Mandic et al., 2015). However, the tasks such as the BPT, due to a limited number of kinematic degrees of freedom (Cormie et al., 2007), do not allow for a marked adaptation of the movement pattern to the altered external load and, thus, could be more suitable for the assessment of the training effects.

Note that this study stems from our previous study (Djuric et al., 2016), where we explored the effect of the training against predominantly constant, inertial, and combined resistance (weight + inertia) through the linear regression model of the force-velocity (F–V) relationship of the muscles performing BPT. BPT was selected for its ballistic properties (characterized by a constant increase in movement velocity), thus mimicking the number of movements typical for sporting activities (e.g., throwing and punching). We found that training of BPT with different types of resistance could result in selective effects on the gains in maximum force, velocity, and power assessed by the F–V relationship parameters. However, the same study did not provide the data regarding the effects of different types of training on the strength (i.e., maximum muscle force) and power produced in the same resistance-specific tasks. Namely, it remains underexplored whether the type (i.e., the constant, inertial, or combined) of the training resistance particularly benefits the movement tasks involving the same resistance type. In addition, the power-producing capacities were assessed only by their maximum values although it has been suggested that the approach based on mechanical outputs averaged over the entire upper limb extension movement could be more representative regarding the analyzed muscular effort (Samozino et al., 2012, 2014).

To address the unanswered questions from the previous study, we aimed to investigate the resistance-specific gains in muscle strength and power-producing capacities following the training of maximum BPT against the constant, inertial, and combined resistance. Based on the findings of our previous study (Djuric et al., 2016), our first hypothesis was that all three experimental groups would reveal gains in both power and strength. Note that the increase in power depends on increases in both force and velocity (power = force × velocity), so any increase in force or velocity will result in an increase in power, while the increase in force is independent of velocity. Therefore, our second hypothesis was that relative gain in power would be larger than the gain in strength. Finally, based on the distinctive kinematic and kinetic pattern of BPT performed against different types of resistances, our third hypothesis was that the observed gains in power and strength would be resistance specific. The findings were expected to advance our understanding of the potential resistance-specific gains in power and force originating from mechanical properties of the applied resistance types that could be of apparent importance for refining both the training and rehabilitation procedures.

## Materials and Methods

### Participants

The sample size was estimated with respect to the effects of a similar range of loads applied in previous studies involving BPT (Leontijevic et al., 2013; Sreckovic et al., 2015). For an alpha level of 0.05 and power 0.80, the sample sizes from three to nine appeared to be necessary to detect the significant loading effects on power and force capacities. Nevertheless, because of planned multiple comparisons, 48 male physical education students of the mean of 20.5 ± 2 years of age (mean ± SD) were recruited for this study. The standard academic curriculum of participants included six to eight activity classes per week (with both low- and high-intensity exercises), and they could be classified as highly physically active according to the standard questionnaire (Rodríguez-Muñoz et al., 2017). Moreover, they had previous experience in resistance training. Participants were randomly assigned to the following groups: constant resistance group (Const-G), inertial resistance group (Inert-G), combined resistance group (Comb-G), and control group (Contr-G). There were no significant differences in body mass, body height, percent body fat, and 1RM among the groups (as shown in Table 1 for details). Only the participants who were not active athletes, and without chronic diseases, recent injuries, or cardiovascular problems were included in the study. They were instructed to avoid any additional strenuous workout over the course of the study. A detailed explanation regarding the potential risks associated with the applied testing and training protocol was provided, and all participants signed written informed consent. Both the consent and the conducted experimental protocol were in accordance with the Declaration of Helsinki and approved by the Institutional Review Board.

**TABLE 1:** Participants groups.

Group	*N*	BM (kg)	BH (cm)	PBF (%)
Const-G	12	75.2 ± 9.1	180.3 ± 7.8	11.2 ± 5.0
Inert-G	12	76.7 ± 14.4	180.4 ± 9.0	12.9 ± 4.0
Comb-G	12	76.7 ± 6.7	183.7 ± 3.0	9.8 ± 3.0
Contr-G	12	77.9 ± 7.8	181.9 ± 5.5	10.6 ± 3.6

*Data as mean ± SD. G-Const, constant resistance group; Inert-G, inertial resistance group; Comb-G, combined resistance group; Contr-G, control group; BM, body mass; BH, body height; PBF, percent body fat.*

### Experimental Protocol

**FIGURE 1:**
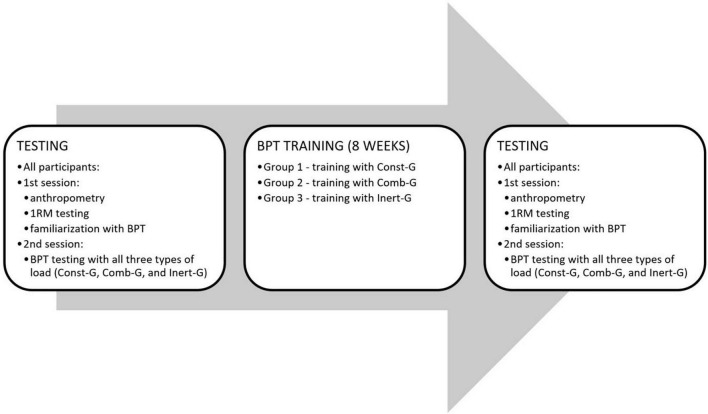
Testing and training protocol. Note that testing and training bench-press throws (BPT) load corresponded to 40 kg of load.

The protocol comprised of the pretest, 8-weeks of training intervention conducted on the three experimental groups, but not on the Cont-G, and the posttest. The pretest consisted of two testing sessions. The first one included anthropometric measurement, the assessment of the muscle strength assessed as 1RM, and a familiarization with loaded BPT (Figure 1). The body mass and body height of participants were assessed with a digital scale and a standard anthropometer, respectively, while their percent body fat was assessed using a bioelectric impedance method (In Body 720; United States). The second session was used to test BPT performed against three resistance types. The first and second sessions of the posttest included the testing of 1RM and BPT, respectively, without any familiarization. A standard 10-min stretching and warm-up procedure consisting of arm and shoulder mobilization exercises and bench press repetitions performed against moderate load was conducted prior to each session (Leontijevic et al., 2013; Sreckovic et al., 2015).

### Training Procedure

Since the maximum power in bench press could be produced against the load corresponding to approximately 50% of 1RM (Baker et al., 2001; Sreckovic et al., 2015), while the average across the participant 1RM was approximately 80 kg, we selected all three types of resistance to corresponding to the different effects of mass of 40 kg. It is important to stress that all three types of resistive forces shared the same *reference load* originating from approximately 10 kg of mass of both the bar and the involved arm segments (Djuric et al., 2016). Since the tested movement was performed in the vertical direction, the reference load provided the combined (i.e., both the constant and inertial) resistance. The remaining part of the load corresponded to three distinctive resistance types, each equivalent to 30 kg of mass. Additional information on the training procedure can be found in our previous publication from which this study originated (Djuric et al., 2016).

**FIGURE 2:**
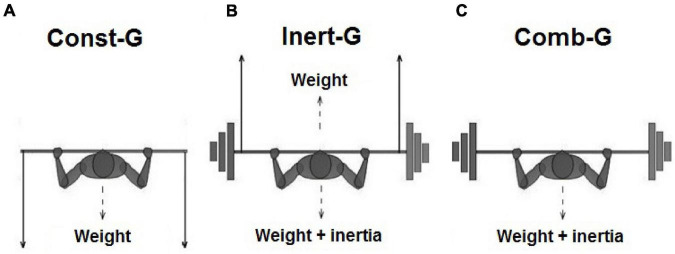
Illustration of three types of training loads. All three types of the resistive forces shared the same “reference load” of approximately 10 kg of mass (the mass of bar and arm segments), while the remaining equivalent of 30 kg originated either from the approximately constant force of stretched rubber bands that mimicked weight (Const-G; panel **A**) or from 30 kg of weight plates that increased both the weight and inertia (Comb-G; panel **B**) or by inertia of the same plates whose weight was compensated by the force of rubber bands pulling upward (Inert-G; panel **C**). The indicated rubber bands forces were adjusted at the starting position. Note that the rest between the sets was 5 min, while the rest between consecutive trials was about 5 s.

To provide three distinctive types of resistive forces for the training and testing purposes, we used a previously employed elaborate combination of the weight plates with the attached rubber bands to the bar of a modified Smith machine (for more details see Leontijevic et al., 2013 and Djuric et al., 2016). Specifically, long rubber bands pulling downward with an approximately constant force of 300 N provided the constant resistance in Const-G (Figure 2A). Namely, since the bands were heavily pre-stretched, the change in their stretched length was only about 6% over the course of the movement yielding a similar change in the resistive force. To provide the inertial resistance for Inert-G, we added 30 kg of weight plates to the bar, while their weight was compensated with rubber bands pulling upward with a resistive force of approximately 300 N (Figure 2B). Finally, a simple addition of weight plates provided a combined resistance for Comb-G (Figure 2C) consisting of both the constant and inertial resistance. As a final result, out of the total equivalent load of 40 kg, all three groups were trained against 10 kg of the load with the property of the combined resistance, while the remaining 30 kg were group-specific. The resistive forces produced by rubber bands were controlled by a calibrated strain gauge attached to the barbell. Specifically, the barbell was attached to the fixed additional bar over a calibrated strain-gauge F transducer (Hottinger, Type S9, range 10 kN; linearity better than 1%, tensile/compressive F sensitivity 2 mV⋅N21) that provided a precise measurement of the resistive forces.

**FIGURE 3:**
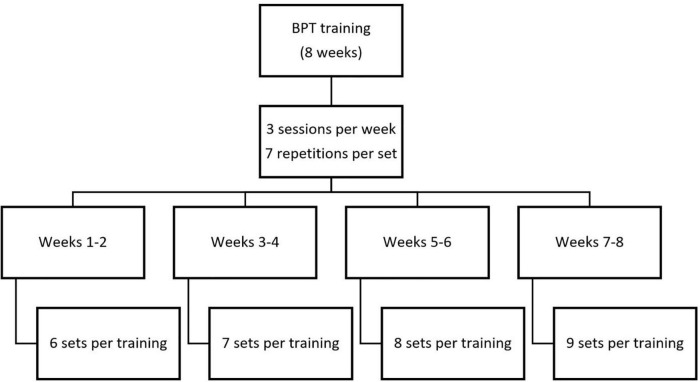
Structure of the 8-week BPT training.

The supervised BPT training was conducted over a period of 8 weeks, three sessions per week. The sessions lasted for about an hour and were preceded by a standardized warm-up and stretching protocol as described above. The number of repetitions and sets is provided in Figure 3. The rest period between the repetitions was 5 s and between the sets 5 min. The exercise (BPT) and training protocol were selected based on the literate review (Baker et al., 2001; Sparkes and Behm, 2010; Hermassi et al., 2011; Kikuchi and Nakazato, 2017). The participants were instructed and verbally encouraged to throw the bar as high as possible, while the Contr-G participants were advised to maintain their regular daily activities throughout the study. Grip width was scaled according to the anthropometric characteristics of participants, with shoulders at 90° of abduction and elbow flexion that provide forearm position mostly perpendicular to the barbell (as shown in Figure 2 for details).

### Testing Procedure

Independently of the applied training resistance, we tested all four participant groups on BPT during the pretest and posttest performed against all three types of resistance applied during the training. The participants completed sets of three trials under each of three resistance types applied in random sequence. The trial with the highest power output was used for further analysis. The sequence of resistances was randomized across the participants, but kept the same in the pretest and posttest in each participant. Both tests also included the assessment of 1RM conducted on the Smith machine according to the standard procedure (Newton et al., 1997; Leontijevic et al., 2013).

### Data Analysis

The bar displacement (distance over time) was recorded by a linear transducer (Vivis Sport Med, Belgrade, Serbia) with an accuracy of 0.01 cm and linearity higher than 99%. The linear transducer has acceptable reliability for testing BPT with ICC ranging from 0.73 to 0.98 and CV% ranging from 2.3 to 6.9 for all F–V relationship parameters (Sreckovic et al., 2015). Data were sampled at a rate of 200 Hz and low-pass filtered using the recursive Butterworth filter with a cutoff frequency of 5 Hz. Two successive derivations of the displacement data provided the velocity and acceleration. Knowing the acceleration and the inertia of the entire system consisting of the sum of weight and inertia of the arm limbs, bar, and depending on the resistance type weight plates or the force exerted against the rubber bands or the combination of the previous two, all other dynamical variables could be calculated. The product of force and velocity over the course of the concentric movement phase provided the muscle power output calculated as both the averaged across the concentric phase and maximum power. The concentric phase was initiated by the first discernible movement of the bar and lasted until the power again reached zero. The instant of the phase termination coincided either with the highest position of the bar (no flight phase was recorded when the constant resistance was applied) or with losing the contact between the hands and the bar (when the inertial and combined resistance was applied) (Cuk et al., 2014; Sreckovic et al., 2015; Djuric et al., 2016; Zivkovic et al., 2017a,b; Janicijevic et al., 2019).

### Statistical Analyses

None of the dependent variables significantly deviated from the normal distribution (Kolmogorov–Smirnov test). The descriptive statistics were calculated as mean and standard deviation. The dependent *t*-test and the Cohen’s *d* (Cohen, 1988) effect size [trivial (0–0.19), small (0.20–0.49), medium (0.50–0.79), and large (0.80 and greater)] were used to assess the effect of training on the power output and strength variables. Since we did not observe any meaningful differences between the pretest and posttest data of Contr-G (all *P* > 0.1), we conducted the subsequent comparison of absolute gains in the maximum and average power, and the strength outputs only on three experimental groups. Wilcoxon’s test was applied to compare individual relative gains (percent changes from pretest to posttest) in power with the same gains in strength. Two-way between-within ANOVA was employed to assess the main effects of “group” (i.e., Const-G, Inert-G, and Comb-G) and “resistance type” (constant, inertial, and combined), and their interaction on power output (*P*_*avg*_ and *P*_*max*_). Due to the limited number of participants, a less conservative Fisher’s least significance difference (LSD) *post-hoc* test was employed to compare the gains in power observed under three resistance types within each group. One-way between ANOVA was applied to compare the gains in strength. Eta squared (*η*^2^) was also calculated together with ANOVA, where the effect sizes 0.01, 0.06, and above 0.14 were considered small, medium, and large, respectively (Cohen, 1988). Alpha was set at *P* = 0.05. All statistical analyses were performed in SPSS for Windows (version 20.0; IBM Corp, Armonk, NY, United States).

## Results

**FIGURE 4:**
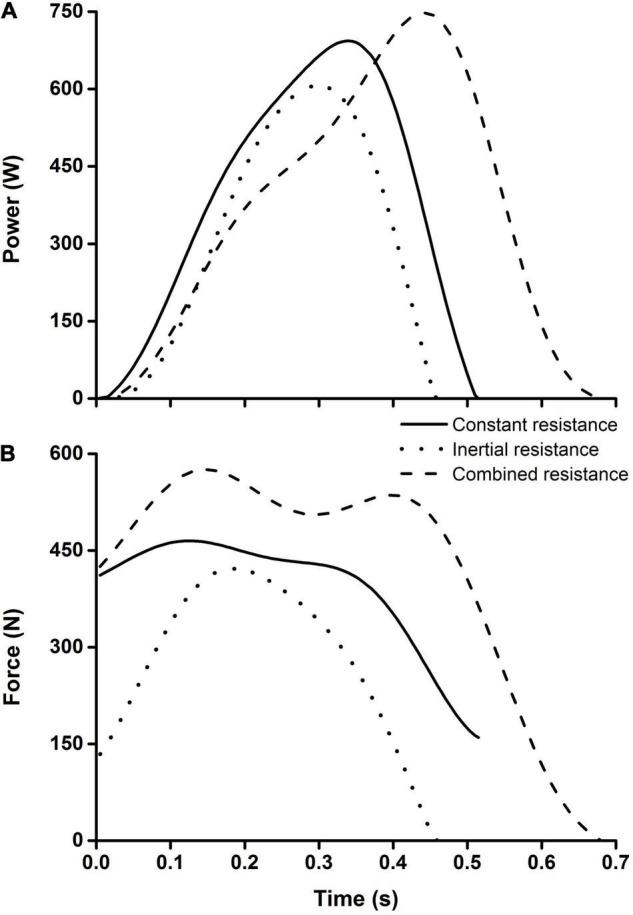
Typical time series of the muscle power **(A)** and strength **(B)** outputs observed from a representative participant.

Figure 4 illustrates typical power and force profiles obtained from trials performed against three resistance types by a representative participant. One can notice that the lowest muscle power and force were observed when acting against the inertial resistance, while the highest force and the longest trial duration were obtained from the combined resistance.

The applied training interventions resulted in marked gains in both the maximum and averaged power in all three experimental groups (as shown in Table 2). The gains ranged from 17 to 36% revealing not only significant differences but also large effect sizes (range 0.90–2.84). Conversely, no meaningful changes in power were observed from Contr-G. As a consequence, Contr-G was excluded from further analysis.

**TABLE 2:** Averaged and maximum power outputs recorded in the pretest and posttest in four participant groups.

		Average power (W)			Maximum power (W)		
Res.	Group	Pretest	Posttest	ES	Pretest	Posttest	ES
Constant	Const-G	388 ± 71	496 ± 60*	1.66	680 ± 142	922 ± 136*	1.75
	Inert-G	417 ± 63	509 ± 65*	1.44	734 ± 124	906 ± 124*	1.39
	Comb-G	402 ± 52	497 ± 31*	2.29	692 ± 98	862 ± 71*	2.01
	Contr-G	427 ± 62	428 ± 60	0.02	758 ± 126	756 ± 119	−0.02
Inertial	Const-G	365 ± 72	434 ± 73*	0.96	633 ± 126	755 ± 147*	0.9
	Inert-G	352 ± 54	466 ± 73*	1.8	625 ± 87	784 ± 120*	1.54
	Comb-G	358 ± 44	441 ± 32*	2.22	610 ± 86	734 ± 65*	1.64
	Contr-G	382 ± 59	377 ± 57	−0.08	657 ± 115	651 ± 107	−0.06
Combined	Const-G	409 ± 90	506 ± 89*	1.09	739 ± 152	914 ± 149*	1.16
	Inert-G	420 ± 83	524 ± 84*	1.25	781 ± 127	916 ± 147*	0.99
	Comb-G	401 ± 47	523 ± 39*	2.85	706 ± 75	901 ± 63*	2.83
	Contr-G	432 ± 74	435 ± 69	0.04	790 ± 121	782 ± 115	−0.06

*Res., resistance type; P_avg_, average power; P_max_, maximum power; ES, effects size.*

*P < 0.01 (different from the pretest)

The changes observed in muscle strength output in all three experimental groups as assessed by 1RM were significant but on average moderate (relative increase 5–9%; ES 0.38–0.75; as shown in Table 3). No change was observed in Contr-G. Although the absolute strength gain was markedly higher in Comb-G (mean 7.5, *s* = 5.0 kg) than that in either Inert-G (mean 3.8, *s* = 5.7 kg) or Const-G (mean 5.4, *s* = 4.0 kg), one-way ANOVA revealed no significant differences among the three experimental groups (*F*_2, 33_ = 1.7; *P* = 0.092; μ_*p*_ = 0.095).

**TABLE 3:** Strength assessed through 1RM (in kg).

Group	Pretest	Posttest	ES
Const-G	80 ± 11	85 ± 12**	0.46
Inert-G	83 ± 11	87 ± 8*	0.39
Comb-G	83 ± 11	90 ± 9**	0.75
Contr-G	82 ± 10	82 ± 10	0.00

*Data are presented as mean ± SD. ES, effect size.*

**P < 0.01 (different from the pretest);

*P < 0.05 (different from the pretest).

*Averaged and maximum power outputs were recorded in the pretest and posttest in four participant groups.*

The individual relative gains averaged across all three resistances in participants of three experimental groups in both *P*_*avg*_ (mean 26.3, *s* = 9.8%) and *P*_*max*_ (mean 25.2, *s* = 9.8%) were compared with the same gains in strength (mean 7.2, *s* = 6.9%). Both differences proved to be significant (Wilcoxon *Z* = 5.1 and *Z* = 5.2, respectively; both *P* < 0.001).

**FIGURE 5:**
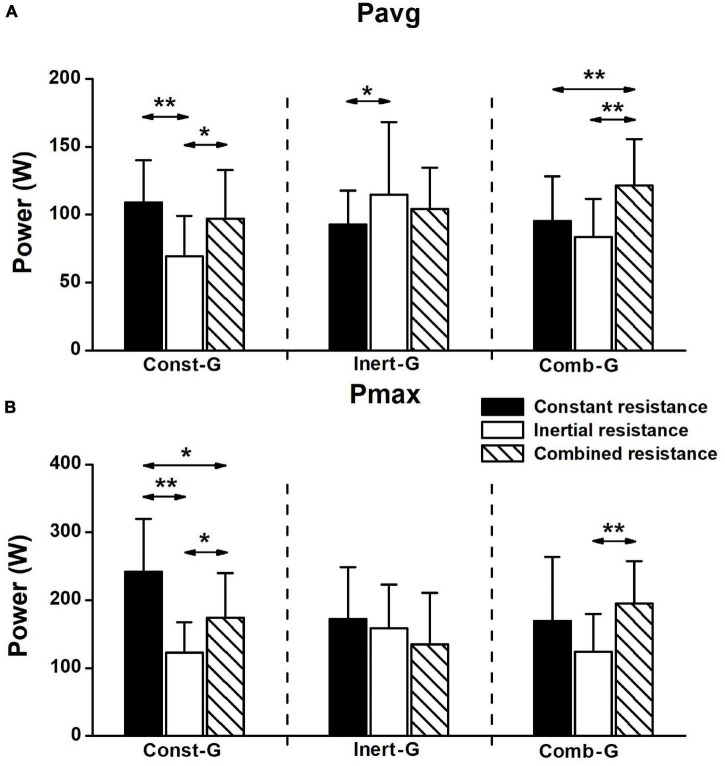
Absolute gains in the averaged (**(A)**; means with *s* error bars) and maximum **(B)** power outputs recorded between the pretest and posttest in three experimental groups under each of three resistance types. Horizontal arrows indicate significant differences between particular resistances observed within the individual groups (LSD; **P < 0.01, *P < 0.05).

The comparisons of the absolute gains in power observed in three experimental groups under different resistance types are shown in Figure 5. Two-way mixed model ANOVA revealed the main effect of resistance type (*F*_2, 66_ = 4.690; *P* < 0.05; μ_*p*_ = 0.124, and *F*_2, 66_ = 9.6; *P* < 0.01; μ_*p*_ = 0.226 for *P*_*avg*_ and *P*_*max*_, respectively), but not of the group (*F*_2, 33_ = 0.6; *P* > 0.05; μ_*p*_ = 0.036, and *F*_2, 33_ = 0.7; *P* > 0.05; μ_*p*_ = 0.041). Of particular importance are the significant interactions observed in *P*_*avg*_ (*F*_4, 66_ = 6.0; *P* < 0.01; μ_*p*_ = 0.265) and *P*_*max*_ (*F*_4, 66_ = 4.7; *P* < 0.01; μ_*p*_ = 0.221). As shown in the figure, the magnitudes of the absolute gains were mainly the training resistance specific. Specifically, seven out of nine significant *post hoc* comparisons suggested that the gains in power were higher when tested against the same resistance applied during the training than when tested against the remaining two resistance types. The remaining two significant differences revealed higher gains observed under the combined than that under the inertial resistance in Const-G.

## Discussion and Implications

Within this study, we explored the effects of training of maximum BPT against the constant resistance, inertial resistance, and their combination on the muscle power, and strength (assessed as 1RM) outputs in young and physically active individuals. The observed results revealed three important findings. In line with our first hypothesis, the applied moderate magnitude of training resistance (i.e., 50% of 1RM) resulted in marked gains in both the muscle power and strength outputs. In line with our second hypothesis, the gains were larger regarding the power than that regarding the strength output. Finally, most of the data supported our third hypothesis regarding the type of resistance-specific effects of the applied training. Namely, higher gains were typically observed under the training resistance than that under the remaining two resistance types.

Of importance for both interpreting, the observed findings and possible applications in training is the specificity of the mechanical properties of applied resistance types. Namely, the constant resistance mimics the action of the gravitational force acting upon the object (or, simply, its weight), the inertial resistance (mimics inertia) mimics the inertial force acting upon the accelerating object (e.g., rapid swings in a predominantly horizontal direction), while the combined resistance is simply the summed up weight and inertia whose vectors are collinear when lifting an object of moderate weight vertically (note that moving heavyweights is inevitably associated with low acceleration). The potential importance of both the weight and inertia in muscle training is reflected even in the advanced resistive exercise device designed by NASA to provide the resistances that separately mimic the effects of constant and inertial resistance (Loehr et al., 2011). Our results suggest that all three applied training resistances could be highly effective in increasing both muscle strength and, particularly, power output. Our finding is in line with several previous studies (McBride et al., 2002; Cormie et al., 2010; Markovic et al., 2013; Djuric et al., 2016) and could be explained by the magnitude of the applied resistances that was about 50% of the muscle strength. Namely, if the F–V relationship of the muscles during BPT (Sreckovic et al., 2015; Djuric et al., 2016) and other multi-joint functional tasks (Cuk et al., 2014; Zivkovic et al., 2017b) is approximately linear, the maximum power output should be *a priori* produced against a resistive force corresponding to 50% of muscle strength (Baker et al., 2001; Siegel et al., 2002; Kraemer and Ratamess, 2004). Moreover, Djuric et al. (2016) revealed that training against a particular load generally increased power due to both the force and velocity increase. That could also explain why the gains in muscle strength were relatively small, as compared to the gains in the power output, since the gains in velocity that in turn contribute to power gains were not assessed. It should be also kept in mind that the highest gains in muscle strength are typically associated with the training against sub-maximum resistances (Cormie et al., 2010; Helms et al., 2016), while that applied in this study was only about 50% of 1RM. Therefore, it is plausible to assume that the observed prominent gains in power were more based on the gains in the muscle maximum velocity than on the gain in the muscle strength output.

Although all three applied resistance types nominally corresponded to the load of 40 kg, Figure 4 suggests that they resulted in prominently different power and force profiles. Similar to the data observed from several of our recent studies (Leontijevic et al., 2013; Markovic et al., 2013; Djuric et al., 2016), the inertial resistance was associated with the lowest power and strength outputs and also resulted in the fastest movements, while the opposite was true for the combined resistance. Nevertheless, we observed no differences regarding the training associated gains in both averaged and maximum power, as well as in the strength outputs among the experimental groups that trained against the three resistances types. This finding is corroborated by our recent study based on the same training intervention (Djuric et al., 2016) where the participants were tested against a wide range of combined resistances that allowed for modeling the muscle F–V relationships.

While the overall (i.e., averaged across the resistances) gains in power did not reveal significant differences in power outputs across the groups, most of the data did reveal the training-specific gains. Specifically, the experimental groups typically revealed the highest gain when tested against the same resistance type as used in the training. Here we could only speculate on the mechanisms involved in the discussed phenomenon. In general, the training-specific gain in muscle capacities is a well-known phenomenon in both training and rehabilitation (Kaneko, 1983; McBride et al., 2002; Kraemer and Ratamess, 2004; Markovic et al., 2013; Djuric et al., 2016). Note that the tested task does not allow for a marked adaptation of the movement pattern to the altered external load that could confound the recorded mechanical variables, such as those observed from loaded vertical jumps (Markovic et al., 2013; Bobbert, 2014; Cuk et al., 2014). Therefore, the improvement in BPT skills was not likely to play the role. However, a plausible explanation could be based on the specific role of the agonist and antagonist muscles acting against the three load types in rapid movements. Namely, it has been known for decades that the constant resistance typically requires only the action of the agonist muscles, while a high inertial load requires a substantial involvement of the antagonists as well (Gottlieb et al., 1989). Therefore, the differences in the relative involvement of the antagonistic muscles could partly explain the observed the training load-specific effects of applied load types. Another explanation could be based on the training associated adaptation to different movement velocities when performed against the three load types. Namely, the fastest movements (i.e., the shortest movement time; see Figure 4) were observed when performed against inertial load, while the opposite applies to the combined load. Future studies need to explore the observed phenomenon of the load-specific gains in muscle power, and the addition of electromyography data could shed a light on it.

The major limitation of this study was the reference load, which was 25% of the total load. This means that 75% of the load was different within the different load types. Future studies should use lighter bars made of more sophisticated materials that can support high loads. Another important limitation is that the external load was absolute and the same for each participant, which was due to the complicated calibration of the loading system. In future studies, this load should be adjusted individually for each participant to obtain an optimal load for maximal muscle power output. In addition, limbs were not taken into account when calculating muscle force and power. Since the percentage of limbs was negligible compared with the total mass of the load, we believe that this did not bias the results obtained to a great extent. Moreover, a force plate could be used to measure the force and not calculate the force indirectly from acceleration and mass. Another limitation of this study is the lack of data on muscle mass and fat percentage during the posttest. In this way, it would be possible to obtain very useful information on the effects of training with different types of loads on the aforementioned variables.

## Conclusion

To conclude, our study revealed that even in physically active individuals, a moderate magnitude of training resistance could result in large gains in the muscle power output. The gain in strength was relatively small indicating that other factors, such as an increase in muscle velocity-producing capacities, could have contributed to the increase in power. However, a particularly novel finding was based on the applied methodological innovation discerning among the three load types. Specifically, although none of the three applied resistance types revealed an overall advantage over the others regarding the gains in power and strength, the obtained results confirmed the gains in power output to be resistance specific. This finding could have important implications for refining training procedures. Specifically, the training aimed to improve the performance of rapid movements should be predominantly based on the inertia-based resistance type, while the improvement in overcoming large external load over a course of relatively slow movements should be predominantly based on the constant resistance type. Future studies should explore whether the observed training effects are based on the different roles of the antagonist muscle when acting against different resistance types, or different movement velocities, or on other mechanisms.

## Data Availability Statement

The raw data supporting the conclusion of this article will be made available by the authors, without undue reservation.

## Ethics Statement

The studies involving human participants were reviewed and approved by University of Belgrade, Faculty of Sports and Physical Education. The patients/participants provided their written informed consent to participate in this study.

## Author Contributions

All authors listed have made a substantial, direct, and intellectual contribution to the work, and approved it for publication.

## Conflict of Interest

The authors declare that the research was conducted in the absence of any commercial or financial relationships that could be construed as a potential conflict of interest.

## Publisher’s Note

All claims expressed in this article are solely those of the authors and do not necessarily represent those of their affiliated organizations, or those of the publisher, the editors and the reviewers. Any product that may be evaluated in this article, or claim that may be made by its manufacturer, is not guaranteed or endorsed by the publisher.
